# Supplementary material for: Insight into plant cell wall degradation and pathogenesis of Ganoderma boninense via comparative genome analysis
Source: PeerJ. 2019 Dec 18;7:e8065. doi: 10.7717/peerj.8065 (PMC6927665; doi:10.7717/peerj.8065)
Supplement: Table S1 [file peerj-07-8065-s001.docx]

| **Glycoside Hydrolases (GHs)** |  |  |
| --- | --- | --- |
|  |  |  |
| **GHs** | ***G. boninense*** | ***G. lucidum^a^*** |
| **GH1** | 5 | 3 |
| **GH2** | 4 | 3 |
| **GH3** | 18 | 12 |
| **GH4** | 1 |  |
| **GH5** | 28 | 19 |
| **GH6** | 1 | 1 |
| **GH7** | 4 | 3 |
| **GH9** | 1 | 2 |
| **GH10** | 13 | 7 |
| **GH12** | 7 | 3 |
| **GH13** | 12 | 9 |
| **GH15** | 5 | 3 |
| **GH16** | 37 | 36 |
| **GH17** | 1 | 3 |
| **GH18** | 40 | 40 |
| **GH20** | 7 | 6 |
| **GH23** | 1 | 1 |
| **GH25** | 3 | 2 |
| **GH27** | 3 | 6 |
| **GH28** | 12 | 13 |
| **GH30** | 2 | 2 |
| **GH31** | 6 | 6 |
| **GH32** | 1 | 1 |
| **GH35** | 6 | 10 |
| **GH37** | 3 | 2 |
| **GH38** | 1 | 1 |
| **GH43** | 19 | 11 |
| **GH45** |  | 2 |
| **GH47** | 11 | 10 |
| **GH51** | 3 | 2 |
| **GH53** | 1 | 1 |
| **GH55** | 3 | 3 |
| **GH63** | 1 |  |
| **GH71** | 7 | 6 |
| **GH72** | 1 | 1 |
| **GH74** | 3 | 1 |
| **GH76** | 3 | 2 |
| **GH78** | 5 | 5 |
| **GH79** | 14 | 11 |
| **GH85** | 3 | 1 |
| **GH88** | 2 | 1 |
| **GH89** | 4 | 1 |
| **GH92** | 7 | 6 |
| **GH93** | 2 | 2 |
| **GH95** | 3 | 1 |
| **GH105** | 5 | 1 |
| **GH109** | 8 |  |
| **GH115** | 5 | 4 |
| **GH125** | 1 | 1 |
| **GH128** | 8 | 6 |
| **GH131** | 4 |  |
| **GH135** | 2 |  |
| **GH145** | 1 |  |
| **Total GH** | **348** | **273** |
|  |  |  |
|  |  |  |
| **Carbohydrate Esterases (CEs)** |  |  |
|  |  |  |
| CEs | ***G. boninense*** | ***G. lucidum^a^*** |
| **CE1** | 13 | 3 |
| **CE2** | 2 |  |
| **CE4** | 6 | 3 |
| **CE8** | 3 | 3 |
| **CE9** | 2 | 1 |
| **CE10** | 46 |  |
| **CE12** | 4 | 1 |
| **CE14** | 1 |  |
| **CE15** | 1 | 2 |
| **CE16** | 24 | 17 |
| **Total CE** | **102** | **30** |
|  |  |  |
|  |  |  |
| **Polysaccharide Lyases (PLs)** |  |  |
|  |  |  |
| **PLs** | ***G. boninense*** | ***G. lucidum^a^*** |
| **PL8** | 3 | 4 |
| **PL12** | 1 |  |
| **PL14** | 10 | 6 |
| **PL15** | 1 |  |
| **Total PL** | **15** | **10** |
|  |  |  |
|  |  |  |
| **Auxiliary Activities (AAs)** |  |  |
|  |  |  |
| **AAs** | ***G. boninense*** | ***G. lucidum^a^*** |
| **AA1** | 22 | 13 |
| **AA2** | 21 | 8 |
| **AA3** | 53 | 5 |
| **AA4** | 2 |  |
| **AA5** | 12 | 9 |
| **AA6** | 2 | 1 |
| **AA7** | 11 |  |
| **AA8** | 2 | 2 |
| **AA9** | 20 | 15 |
| **Total AA** | **145** | **53** |
|  |  |  |
|  |  |  |
| **Carbohydrate-Binding Modules (CBMs)** | |  |
|  |  |  |
| **CBMs** | ***G. boninense*** | ***G. lucidum^a^*** |
| **CBM1** | 18 | 14 |
| **CBM5** | 8 | 10 |
| **CBM12** | 3 | 1 |
| **CBM13** | 10 | 9 |
| **CBM18** |  | 2 |
| **CBM19** | 4 |  |
| **CBM20** | 5 | 3 |
| **CBM21** | 2 | 2 |
| **CBM32** | 1 |  |
| **CBM35** | 1 |  |
| **CBM43** | 1 | 1 |
| **CBM48** | 2 | 3 |
| **CBM50** | 12 | 8 |
| **Total CBM** | **67** | **53** |
|  |  |  |
|  |  |  |
| **Glycosyltransferases (GTs)** |  |  |
|  |  |  |
| **GTs** | ***G. boninense*** | ***G. lucidum^a^*** |
| **GT1** | 12 | 10 |
| **GT2** | 11 | 12 |
| **GT3** | 1 | 1 |
| **GT4** | 5 | 4 |
| **GT5** |  | 1 |
| **GT8** | 7 | 6 |
| **GT15** | 5 | 3 |
| **GT17** | 1 | 2 |
| **GT20** | 4 | 3 |
| **GT21** | 1 | 1 |
| **GT22** | 2 | 3 |
| **GT24** | 1 | 1 |
| **GT25** |  |  |
| **GT31** |  | 1 |
| **GT32** | 4 | 1 |
| **GT33** | 1 | 1 |
| **GT35** | 2 | 1 |
| **GT39** | 3 | 3 |
| **GT41** | 1 |  |
| **GT48** | 3 | 4 |
| **GT49** | 1 | 1 |
| **GT50** | 1 | 1 |
| **GT57** | 2 | 2 |
| **GT58** | 1 | 1 |
| **GT59** | 1 | 1 |
| **GT65** | 2 |  |
| **GT66** | 1 | 1 |
| **GT69** | 3 | 2 |
| **GT76** | 1 | 2 |
| **GT90** | 1 | 1 |
| **Total GT** | **86** | **70** |

^a^CAZymes information of *G. lucidum* was obtained from Chen et al., 2012 whereby GH61 genes were now categorized into AA9 family.

**Reference**

Chen S, Xu J, Liu C, Zhu Y, Nelson DR, Zhou S, Li C, Wang L, Guo X, Sun Y, Luo H, Li Y, Song J, Henrissat B, Levasseur A, Qian J, Li J, Luo X, Shi L, He L, Xiang L, Xu X, Niu Y, Li Q, Han M V, Yan H, Zhang J, Chen H, Lv A, Wang Z, Liu M, Schwartz DC, Sun C. 2012. Genome sequence of the model medicinal mushroom *Ganoderma lucidum*. Nature Communications 3:913
