# Supplementary material for: Insight into plant cell wall degradation and pathogenesis of Ganoderma boninense via comparative genome analysis
Source: PeerJ. 2019 Dec 18;7:e8065. doi: 10.7717/peerj.8065 (PMC6927665; doi:10.7717/peerj.8065)
Supplement: Table S2 — Comparative genome analysis was carried out by comparing CAZymes dataset of G. boninense NJ3 with previously-reported genome sequence of Ganoderma lucidum (Chen et al., 2012a; Chen et al., 2012b), Melampsora larici-populina (Duplessis et al., 2011), Ustilago maydis (Kamper et al., 2006), Moniliophthora perniciosa FA553 (Mondego et al., 2008), Armillaria solidipes 28-4 (Sipos et al., 2017) and Armillaria mellea DSM 3731 (Collins et al., 2013). Except for G. lucidum, archival CAZymes information of the other referenced fungal genomes was retrieved from Joint Genome Institute MycoCosm portal in May 2018 (Grigoriev et al., 2014) (https://gp-dge6.jgi.doe.gov/programs/fungi/index.jsf). [file peerj-07-8065-s002.docx]

| **Glycoside Hydrolases (GHs)** | | |  |  |  |  |  |
| --- | --- | --- | --- | --- | --- | --- | --- |
|  |  |  |  |  |  |  |  |
| **GH** | ***M. perniciosa*** | ***A. mellea*** | ***A. solidipes*** | ***M. larici-populina*** | ***U. maydis*** | ***G. boninense*** | ***G. lucidum*** |
| **GH1** | 7 | 10 | 11 |  |  | 5 | 3 |
| **GH2** | 4 | 2 | 2 | 4 | 1 | 4 | 3 |
| **GH3** | 24 | 15 | 12 | 3 | 3 | 18 | 12 |
| **GH4** |  |  |  |  |  | 1 |  |
| **GH5** | 39 | 20 | 21 | 31 | 12 | 28 | 19 |
| **GH6** | 3 | 2 | 2 |  |  | 1 | 1 |
| **GH7** | 5 | 4 | 3 | 8 |  | 4 | 3 |
| **GH8** |  |  |  |  | 1 |  |  |
| **GH9** | 2 | 1 | 1 | 1 | 1 | 1 | 2 |
| **GH10** | 9 | 7 | 6 | 6 | 2 | 13 | 7 |
| **GH11** | 3 | 2 | 1 |  | 1 |  |  |
| **GH12** | 5 | 4 | 4 | 5 |  | 7 | 3 |
| **GH13** | 10 | 14 | 15 | 7 | 4 | 12 | 9 |
| **GH15** | 3 | 5 | 4 | 4 | 1 | 5 | 3 |
| **GH16** | 40 | 37 | 37 | 11 | 20 | 37 | 36 |
| **GH17** | 3 | 7 | 6 | 2 | 2 | 1 | 3 |
| **GH18** | 19 | 15 | 16 | 15 | 3 | 40 | 40 |
| **GH20** | 2 | 2 | 2 | 3 | 2 | 7 | 6 |
| **GH23** | 1 | 1 | 1 | 1 | 1 | 1 | 1 |
| **GH24** |  |  |  |  |  |  |  |
| **GH25** | 2 | 1 | 1 |  | 1 | 3 | 2 |
| **GH26** |  |  |  | 10 | 1 |  |  |
| **GH27** | 3 | 6 | 7 | 8 | 1 | 3 | 6 |
| **GH28** | 8 | 25 | 17 | 3 | 1 | 12 | 13 |
| **GH29** | 2 | 3 | 4 |  |  |  |  |
| **GH30** | 6 | 6 | 5 |  | 2 | 2 | 2 |
| **GH31** | 11 | 5 | 6 | 4 | 3 | 6 | 6 |
| **GH32** | 1 |  |  | 2 | 2 | 1 | 1 |
| **GH33** |  |  |  |  |  |  |  |
| **GH35** | 8 | 8 | 5 | 1 | 1 | 6 | 10 |
| **GH36** |  |  |  |  | 1 |  |  |
| **GH37** | 8 | 3 | 3 | 5 | 2 | 3 | 2 |
| **GH38** | 1 | 1 | 1 | 1 | 2 | 1 | 1 |
| **GH39** |  | 1 | 1 |  |  |  |  |
| **GH42** |  | 1 |  |  | 1 |  |  |
| **GH43** | 17 | 8 | 10 | 8 | 4 | 19 | 11 |
| **GH44** | 1 |  |  |  |  |  |  |
| **GH45** | 3 | 3 | 4 | 1 | 4 |  | 2 |
| **GH46** |  |  |  |  |  |  |  |
| **GH47** | 18 | 12 | 9 | 15 | 3 | 11 | 10 |
| **GH50** |  |  |  |  |  |  |  |
| **GH51** | 4 | 3 | 4 | 3 | 2 | 3 | 2 |
| **GH53** | 1 | 2 | 2 |  |  | 1 | 1 |
| **GH54** |  |  |  |  |  |  |  |
| **GH55** | 13 | 4 | 6 |  | 1 | 3 | 3 |
| **GH62** |  |  |  |  | 1 |  |  |
| **GH63** |  | 1 | 1 | 1 | 1 | 1 |  |
| **GH64** |  |  |  |  |  |  |  |
| **GH65** |  |  |  |  |  |  |  |
| **GH67** |  |  |  |  |  |  |  |
| **GH71** | 4 | 7 | 7 | 5 |  | 7 | 6 |
| **GH72** | 1 | 1 | 1 | 1 | 1 | 1 | 1 |
| **GH74** | 3 | 2 | 2 |  |  | 3 | 1 |
| **GH75** | 2 | 2 | 2 |  |  |  |  |
| **GH76** | 2 | 3 | 5 | 3 | 1 | 3 | 2 |
| **GH78** | 1 | 3 | 4 |  |  | 5 | 5 |
| **GH79** | 7 | 10 | 10 | 2 | 1 | 14 | 11 |
| **GH81** |  |  |  | 2 |  |  |  |
| **GH85** | 1 | 1 | 1 | 2 | 1 | 3 | 1 |
| **GH88** | 4 | 2 | 2 |  |  | 2 | 1 |
| **GH89** |  |  |  |  |  | 4 | 1 |
| **GH92** | 12 | 4 | 4 | 2 | 3 | 7 | 6 |
| **GH93** |  | 1 | 1 |  |  | 2 | 2 |
| **GH94** |  |  |  |  |  |  |  |
| **GH95** | 7 | 1 | 2 |  |  | 3 | 1 |
| **GH105** | 5 | 2 | 2 |  | 1 | 5 | 1 |
| **GH106** |  |  |  |  |  |  |  |
| **GH109** |  |  |  |  |  | 8 |  |
| **GH114** | 1 |  |  |  |  |  |  |
| **GH115** | 6 | 2 | 4 |  | 1 | 5 | 4 |
| **GH125** | 1 | 1 | 1 |  | 1 | 1 | 1 |
| **GH127** |  |  |  |  |  |  |  |
| **GH128** | 3 | 7 | 6 |  | 5 | 8 | 6 |
| **GH130** |  |  |  |  |  |  |  |
| **GH131** | 3 | 1 | 3 | 9 |  | 4 |  |
| **GH132** |  |  |  |  |  |  |  |
| **GH133** | 2 | 1 | 1 | 1 | 1 |  |  |
| **GH135** | 1 | 1 | 1 |  | 1 | 2 |  |
| **GH145** |  | 1 |  |  |  | 1 |  |
| **GH152** |  | 5 |  |  |  |  |  |
| **Total GH** | **352** | **299** | **289** | **190** | **105** | **348** | **273** |
|  |  |  |  |  |  |  |  |
|  |  |  |  |  |  |  |  |
| **Carbohydrate Esterases (CEs)** | | |  |  |  |  |  |
|  |  |  |  |  |  |  |  |
| **CE** | ***M. perniciosa*** | ***A. mellea*** | ***A. solidipes*** | ***M. larici-populina*** | ***U. maydis*** | ***G. boninense*** | ***G. lucidum*** |
| **CE1** | 2 | 1 | 1 |  | 1 | 13 | 3 |
| **CE2** |  |  |  |  |  | 2 |  |
| **CE4** | 20 | 16 | 16 | 18 | 7 | 6 | 3 |
| **CE5** | 2 |  |  | 7 | 4 |  |  |
| **CE8** | 2 | 10 | 9 | 5 | 1 | 3 | 3 |
| **CE9** | 2 | 1 | 1 |  | 1 | 2 | 1 |
| **CE10** |  |  |  |  |  | 46 |  |
| **CE12** | 1 | 4 | 4 |  |  | 4 | 1 |
| **CE14** |  |  |  |  |  | 1 |  |
| **CE15** | 1 | 4 | 2 |  |  | 1 | 2 |
| **CE16** | 5 | 11 | 10 | 16 |  | 24 | 17 |
| **Total CE** | **35** | **47** | **43** | **46** | **14** | **102** | **30** |
|  |  |  |  |  |  |  |  |
|  |  |  |  |  |  |  |  |
| **Polysaccharide Lyases (PLs)** | | |  |  |  |  |  |
|  |  |  |  |  |  |  |  |
| **PL** | ***M. perniciosa*** | ***A. mellea*** | ***A. solidipes*** | ***M. larici-populina*** | ***U. maydis*** | ***G. boninense*** | ***G. lucidum*** |
| **PL1** | 15 | 6 | 6 | 4 | 1 |  |  |
| **PL3** | 4 | 9 | 12 |  |  |  |  |
| **PL4** | 2 | 2 | 2 |  |  |  |  |
| **PL8** |  | 3 | 3 |  |  | 3 | 4 |
| **PL12** |  |  |  |  |  | 1 |  |
| **PL14** | 1 | 4 | 4 | 2 |  | 10 | 6 |
| **PL15** |  |  |  |  |  | 1 |  |
| **PL26** |  | 1 |  |  |  |  |  |
| **Total PL** | **22** | **25** | **27** | **6** | **1** | **15** | **10** |
|  |  |  |  |  |  |  |  |
|  |  |  |  |  |  |  |  |

| **Auxiliary Activities (AAs)** | | |  |  |  |  |  |
| --- | --- | --- | --- | --- | --- | --- | --- |
|  |  |  |  |  |  |  |  |
| **AA** | ***M. perniciosa*** | ***A. mellea*** | ***A. solidipes*** | ***M. larici-populina*** | ***U. maydis*** | ***G. boninense*** | ***G. lucidum*** |
| **AA1** | 30 | 28 | 26 | 6 | 6 | 22 | 13 |
| **AA2** | 4 | 10 | 7 |  |  | 21 | 8 |
| **AA3** | 72 | 67 | 61 | 7 | 9 | 53 | 5 |
| **AA4** |  |  |  |  |  | 2 |  |
| **AA5** | 14 | 7 | 5 | 4 | 4 | 12 | 9 |
| **AA6** | 1 | 3 | 4 | 1 | 1 | 2 | 1 |
| **AA7** |  | 3 | 4 | 14 | 2 | 11 |  |
| **AA8** | 2 | 2 | 2 |  |  | 2 | 2 |
| **AA9** | 18 | 25 | 19 | 4 |  | 20 | 15 |
| **AA10** |  |  |  |  | 1 |  |  |
| **AA12** | 2 |  |  |  |  |  |  |
| **AA14** |  | 4 |  |  |  |  |  |
| **Total AA** | **143** | **149** | **128** | **36** | **23** | **145** | **53** |
|  |  |  |  |  |  |  |  |
|  |  |  |  |  |  |  |  |
| **Carbohydrate-Binding Modules (CBMs)** | | | |  |  |  |  |
|  |  |  |  |  |  |  |  |
| **CBM** | ***M. perniciosa*** | ***A. mellea*** | ***A. solidipes*** | ***M. larici-populina*** | ***U. maydis*** | ***G. boninense*** | ***G. lucidum*** |
| **CBM1** | 5 | 11 | 11 |  |  | 18 | 14 |
| **CBM5** | 3 | 3 | 4 |  |  | 8 | 10 |
| **CBM12** | 1 | 1 | 1 | 1 |  | 3 | 1 |
| **CBM13** | 4 | 7 | 7 |  |  | 10 | 9 |
| **CBM18** | 1 |  |  |  | 2 |  | 2 |
| **CBM19** |  |  | 1 |  |  | 4 |  |
| **CBM20** | 2 | 3 | 5 |  |  | 5 | 3 |
| **CBM21** | 1 | 2 | 2 | 1 |  | 2 | 2 |
| **CBM32** |  |  |  |  |  | 1 |  |
| **CBM35** |  | 1 | 2 | 3 | 1 | 1 |  |
| **CBM43** |  | 1 | 1 | 1 | 1 | 1 | 1 |
| **CBM48** | 2 | 3 | 3 | 3 | 2 | 2 | 3 |
| **CBM50** | 4 | 14 | 21 | 1 | 1 | 12 | 8 |
| **CBM52** |  |  |  |  |  |  |  |
| **CBM63** |  |  |  | 4 |  |  |  |
| **CBM67** | 1 | 2 | 29 |  |  |  |  |
| **Total CBM** | **24** | **48** | **87** | **14** | **7** | **67** | **53** |
|  |  |  |  |  |  |  |  |
|  |  |  |  |  |  |  |  |
| **Glycosyltransferases (GTs)** | | |  |  |  |  |  |
|  |  |  |  |  |  |  |  |
| **GT** | ***M. perniciosa*** | ***A. mellea*** | ***A. solidipes*** | ***M. larici-populina*** | ***U. maydis*** | ***G. boninense*** | ***G. lucidum*** |
| **GT1** | 9 | 11 | 7 | 7 | 5 | 12 | 10 |
| **GT2** | 7 | 13 | 13 | 14 | 14 | 11 | 12 |
| **GT3** | 2 | 1 | 1 | 1 | 1 | 1 | 1 |
| **GT4** | 8 | 7 | 8 | 5 | 4 | 5 | 4 |
| **GT5** | 1 | 1 | 1 | 2 |  |  | 1 |
| **GT8** | 3 | 6 | 6 | 2 | 2 | 7 | 6 |
| **GT10** |  |  |  |  |  |  |  |
| **GT15** | 7 | 6 | 6 | 2 | 2 | 5 | 3 |
| **GT17** | 2 | 1 | 1 |  | 1 | 1 | 2 |
| **GT18** |  |  |  | 2 |  |  |  |
| **GT20** | 5 | 4 | 4 | 3 | 3 | 4 | 3 |
| **GT21** | 1 | 1 | 1 | 1 | 1 | 1 | 1 |
| **GT22** | 4 | 1 | 3 | 4 | 4 | 2 | 3 |
| **GT24** | 1 | 1 | 1 | 1 | 1 | 1 | 1 |
| **GT25** |  |  |  | 1 |  |  |  |
| **GT31** | 1 | 1 | 1 | 6 | 3 |  | 1 |
| **GT32** | 1 | 2 | 3 | 6 | 3 | 4 | 1 |
| **GT33** | 1 | 1 | 1 | 1 | 1 | 1 | 1 |
| **GT35** | 4 | 2 | 1 |  |  | 2 | 1 |
| **GT39** | 6 | 4 | 3 | 3 | 3 | 3 | 3 |
| **GT41** |  |  |  | 2 | 1 | 1 |  |
| **GT43** |  |  |  |  |  |  |  |
| **GT48** | 9 | 2 | 2 | 8 | 1 | 3 | 4 |
| **GT49** | 1 | 1 | 1 | 1 |  | 1 | 1 |
| **GT50** | 1 | 1 | 1 | 1 | 1 | 1 | 1 |
| **GT57** | 4 | 1 | 2 | 2 | 2 | 2 | 2 |
| **GT58** | 2 | 1 | 1 | 3 | 1 | 1 | 1 |
| **GT59** |  | 1 | 1 | 1 | 1 | 1 | 1 |
| **GT65** |  |  |  |  |  | 2 |  |
| **GT66** |  | 1 | 1 | 1 | 1 | 1 | 1 |
| **GT69** | 1 | 4 | 3 | 1 | 3 | 3 | 2 |
| **GT71** |  |  |  | 2 | 2 |  |  |
| **GT76** |  |  | 1 | 1 | 1 | 1 | 2 |
| **GT90** | 2 | 5 | 4 | 9 | 2 | 1 | 1 |
| **Total GT** | **83** | **80** | **78** | **93** | **64** | **78** | **70** |

**References**

Chen S, Xu J, Liu C, Zhu Y, Nelson DR, Zhou S, Li C, Wang L, Guo X, Sun Y, Luo H, Li Y, Song J, Henrissat B, Levasseur A, Qian J, Li J, Luo X, Shi L, He L, Xiang L, Xu X, Niu Y, Li Q, Han M V, Yan H, Zhang J, Chen H, Lv A, Wang Z, Liu M, Schwartz DC, Sun C. 2012b. Genome sequence of the model medicinal mushroom *Ganoderma lucidum*. *Nature Communications* 3:913.

Collins C, Keane TM, Turner DJ, O’Keeffe G, Fitzpatrick DA, Doyle S. 2013. Genomic and proteomic dissection of the ubiquitous plant pathogen, *Armillaria mellea*: Toward a new infection model system. *Journal of Proteome Research*. 12(6):2552-70

Duplessis S, Cuomo CA, Lin YC, Aerts A, Tisserant E, Veneault-Fourrey C, Joly DL, Hacquard S, Amselem J, Cantarel BL, Chiu R, Coutinho PM, Feau N, Field M, Frey P, Gelhaye E, Goldberg J, Grabherr MG, Kodira CD, Kohler A, Kues U, Lindquist EA, Lucas SM, Mago R, Mauceli E, Morin E, Murat C, Pangilinan JL, Park R, Pearson M, Quesneville H, Rouhier N, Sakthikumar S, Salamov AA, Schmutz J, Selles B, Shapiro H, Tanguay P, Tuskan GA, Henrissat B, Van de Peer Y, Rouze P, Ellis JG, Dodds PN, Schein JE, Zhong S, Hamelin RC, Grigoriev IV, Szabo LJ, Martin F. 2011. Obligate biotrophy features unraveled by the genomic analysis of rust fungi. *Proceedings of the National Academy of Sciences*. 108(22):9166-71

Grigoriev IV, Nikitin R, Haridas S, Kuo A, Ohm R, Otillar R, Riley R, Salamov A. Zhao X, Korzeniewski F, Smirnova T, Nordberg H, Dubchak I, Shabalov I. 2014. MycoCosm portal: gearing up for 1000 fungal genomes. *Nucleic Acids Research* 42:D699–D704.

Kämper J, Kahmann R, Bölker M, Ma LJ, Brefort T, Saville BJ, Banuett F, Kronstad JW, Gold SE, Müller O, Perlin MH, Wösten HAB, de Vries R, Ruiz-Herrera J, Reynaga-Peña CG, Snetselaar K, McCann M, Pérez-Martín J, Feldbrügge M, Basse CW, Steinberg G, Ibeas JI, Holloman W, Guzman P, Farman M, Stajich JE, Sentandreu R, González-Prieto JM, Kennell JC, Molina L, Schirawski J, Mendoza-Mendoza A, Greilinger D, Münch K, Rössel N, Scherer M, Vraneš M, Ladendorf O, Vincon V, Fuchs U, Sandrock B, Meng S, Ho ECH, Cahill MJ, Boyce KJ, Klose J, Klosterman SJ, Deelstra HJ, Ortiz-Castellanos L, Li W, Sanchez-Alonso P, Schreier PH, Häuser-Hahn I, Vaupel M, Koopmann E, Friedrich G, Voss H, Schlüter T, Margolis J, Platt D, Swimmer C, Gnirke A, Chen F, Vysotskaia V, Mannhaupt G, Güldener U, Münsterkötter M, Haase D, Oesterheld M, Mewes HW, Mauceli EW, DeCaprio D, Wade CM, Butler J, Young S, Jaffe DB, Calvo S, Nusbaum C, Galagan J, Birren BW. 2006. Insights from the genome of the biotrophic fungal plant pathogen *Ustilago maydis*. *Nature* 444:97–101.

Mondego JMC, Carazzolle MF, Costa GGL, Formighieri EF, Parizzi LP, Rincones J, Cotomacci C, Carraro DM, Cunha AF, Carrer H, Vidal RO, Estrela RC, García O, Thomazella DPT, de Oliveira BV, Pires ABL, Rio MC, Araújo MRR, de Moraes MH, Castro LAB, Gramacho KP, Gonçalves MS, Neto JPM, Neto AG, Barbosa LV, Guiltinan MJ, Bailey BA, Meinhardt LW, Cascardo JCM, Pereira GAG. 2008. A genome survey of *Moniliophthora perniciosa* gives new insights into Witches’ Broom Disease of cacao. *BMC Genomics*. 9:548

Sipos G, Prasanna AN, Walter MC, O’Connor E, Bálint B, Krizsán K, Kiss B, Hess J, Varga T, Slot J, Riley R, Bóka B, Rigling D, Barry K, Lee J, Mihaltcheva S, Labutti K, Lipzen A, Waldron R, Moloney NM, Sperisen C, Kredics L, Vágvölgyi C, Patrignani A, Fitzpatrick D, Nagy I, Doyle S, Anderson JB, Grigoriev IV, Güldener U, Münsterkötter M, Nagy LG. 2017. Genome expansion and lineage-specific genetic innovations in the forest pathogenic fungi *Armillaria*. *Nature Ecology and Evolution*. 1(12):1931-1941
